# Identification of biomarkers and drug repurposing candidates based on an immune-, inflammation- and membranous glomerulonephritis-associated triplets network for membranous glomerulonephritis

**DOI:** 10.1186/s12920-019-0655-8

**Published:** 2020-01-07

**Authors:** Chengwei Zhang, Lei Leng, Zhaozheng Li, Yao Zhao, Jundong Jiao

**Affiliations:** 1 Department of nephrology, the Second Affiliated Hospital of Harbin Medical University, 246 XueFu Road, Harbin, 150006 People’s Republic of China; 2 The Second Hospital of Harbin, Heilongjiang, 150006 People’s Republic of China

**Keywords:** Drug repurposing, Biomarker, Immune response, Inflammation response, Membranous glomerulonephritis

## Abstract

**Background:**

Membranous glomerulonephritis (MGN) is a common kidney disease. Despite many evidences support that many immune and inflammation-related genes could serve as effective biomarkers and treatment targets for MGN patients, the potential associations among MGN-, immune- and inflammation-related genes have not been sufficiently understood.

**Methods:**

Here, a global immune-, inflammation- and MGN-associated triplets (IIMATs) network is constructed and analyzed. An integrated and computational approach is developed to identify dysregulated IIMATs for MGN patients based on expression and interaction data.

**Results:**

45 dysregulated IIMATs are identified in MGN by above method. Dysregulated patterns of these dysregulated IIMATs are complex and various. We identify four core clusters from dysregulated IIMATs network and some of these clusters could distinguish MGN and normal samples. Specially, some anti-cancer drugs including Tamoxifen, Bosutinib, Ponatinib and Nintedanib could become candidate drugs for MGN based on drug repurposing strategy follow IIMATs. Functional analysis shows these dysregulated IIMATs are associated with some key functions and chemokine signaling pathway.

**Conclusions:**

The present study explored the associations among immune, inflammation and MGN. Some effective candidate drugs for MGN were identified based on immune and inflammation. Overall, these comprehensive results provide novel insights into the mechanisms and treatment of MGN.

## Background

Membranous glomerulonephritis (MGN) is a slowly progressive immune and inflammation-associated disease of the kidney affecting mostly adult population [1, 2]. MGN usually shows the thickening of the glomerular basement membrane in the kidney tissue [3]. Recent studies have revealed that most MGN patients have IgG4 antibodies to the phospholipase A2 receptor (PLA2R) [4, 5]. Renal biopsy and the detection of antibodies to the few podocyte antigens are two common current diagnostic methods. However, the global and detail mechanism and etiology of MGN are unknown because of the limitations of the current diagnostic approaches, including invasiveness and the lack of sensitivity of the current biomarkers. Therefore, there is a requirement to identify more novel applicable biomarkers and signatures that can enhance clinical behaviors in the treatment of MGN.

Some previous studies have revealed that there were close associations among MGN, inflammation and immune. Inflammation is usually known as a homeostatic phenomenon, which is induced by various inflammatory agents. Chronic inflammation is one of major characteristic for MGN [6]. An inflammatory transcription factor named Nuclear factor-kappa B (NF-KB) is over-expressed in patients with MGN [7]. The dysregulation of inflammatory response could lead the body to heal from the tissue lesion [8]. MGN also usually occurred because of immunoglobulin and associated with other autoimmune conditions [9, 10]. Although these findings all demonstrated that MGN is associated with immune and inflammation, the detailed and global mechanism and relationships for immune and inflammation in MGN is unknown.

In MGN patients, prospective randomized clinical trials have demonstrated that the calcineurin inhibitors cyclosporine [11] and tacrolimus [12] induce complete or partial remission of proteinuria in more than 70% of patients. However, more than 60% of patients treated with calcineurin inhibitors suffer subsequent relapses or become treatment dependent and need prolonged therapy to maintain remission, which exposes them to the nephrotoxic effects of this drugs. Consequently, for these patients, there is a need for the development [13] of new treatment strategies aimed at reducing the risk of chronic nephrotoxicity. Research into and development of novel drugs for MGN is time-consuming and labour-intensive processes. Recent years, drug repurposing, an essential and successful drug discovery could discover the novel indications of existing drugs. There are a number of successful examples such as Pfizer’s Viagra for erectile dysfunction and Celgene’s thalidomide for leprosy and multiple myeloma based on drug repurposing [14, 15]. However, few studies have focused on drug repurposing for MGN, especially based on immune response and inflammation.

In present study, we first construct a global immune-, inflammation- and MGN-associated triplets (IIMATs) network and analyze its topological features. Some dysregulated IIMATs are also identified in MGN patients. The risk score profiles and dysregulated patterns of IIMATs are varied in MGN patients. We also find some MGN related core clusters could as specific biomarker to distinguish MGN and matched control samples. Specially, novel drug repurposing candidates for MGN are identified based on IIMATs. Moreover, MGN-related IIMATs are associated with critical biological functions and the chemokine signalling pathway. These comprehensive results could provide novel insights into the underlying mechanisms and treatment of MGN.

## Methods

### Human immune and inflammation-associated gene datasets

The immune and inflammation-associated genes are downloaded from AmiGO 2 version: 2.4.26 (2018-04-22, http://amigo2.berkeleybop.org/amigo) [16] in *Homo sapiens* species, consisting 3014 immune-related genes from 651 records and 604 inflammation-related genes from 91 records (Additional file 1: Table S1).

### MGN-associated gene datasets

All the MGN-associated gene are obtained from database DisGeNET which is a public platform collecting genes associated with various kinds of human diseases [17]. Finally, 90 MGN-associated genes are included in our follow analysis (Additional file 1: Table S1).

### Construction of IIMAT network based on human protein-protein interaction data and topological feature analysis

We obtain protein-protein interaction (PPI) data from the HPRD (Human Protein Reference Database, http://www.hprd.org/, Release 9, 2010-4-13) database [18]. HPRD is a database of curated proteomic information pertaining to human proteins. Lastly, 39,240 PPIs were included for follow analysis. It is defined as an IIMAT when MGN, immune and inflammation-associated gene exist interaction based on PPI network. Thus the interactions including immune-inflammation, immune-MGN and inflammation-MGN were discovered. Then an IIMAT network is constructed based on above three kinds of interactions, topological features and R-square of degree are analyzed using Cytoscape 3.3.0 (http://www.cytoscape.org/).

### Collection of high-throughput gene expression data

The gene expression profiles for MGN are downloaded from Gene Expression Omnibus (GEO) database (www.ncbi.nlm.nih.gov/geo). There are 21 MGN patients and 18 control samples in the gene expression profile data (GSE99340) [19]. Demographic data of these 21 patients are provided in Additional file 2: Table S2. Other detailed information could be found in a previous study and public database GEO. Probe ids information Affymetrix Human Genome U133A Array was downloaded from platform GPL19184 (https://www.ncbi.nlm.nih.gov/geo/query/acc.cgi?acc=GPL19184). Average values would be represented as gene expression if multiple probe ids matched to a same gene name.

### Identification of MGN-specific IIMATs based on expression data and network

A comprehensive and calculational method is developed to identify MGN-specific IIMATs based on gene expression and network data. First of all, Student’ s t-test are used to compare differences in gene expression between MGN patients and the matched controls for each IIMAT. Second, for gene interaction in each IIMAT, Pearson’s correlation coefficients (PCCs) are computed in MGN and matched samples, respectively. We use absolute values of difference for the PCCs between MGN and matched samples to represent the change of interactions from normal to disease. Two integrated scores

*Score*_*dif*_*and Score*_*PCC*_ are designed to estimate dysregulated level each IIMAT in MGN patients. The detailed equations for the two integrated scores are shown as follows:

$$ {\displaystyle \begin{array}{l} Scor{e}_{dif}={P}_{im mune}\ast {P}_{\operatorname{inf} lammation}\ast {P}_{MGN}\\ {} Scor{e}_{PCC}=\mid \left({\mathrm{MGN}}_{im- in}- Norma{l}_{im- in}\right)\ast \left( MG{N}_{im- MGN}- Norma{l}_{im- MGN}\right)\ast \left( MG{N}_{in- MGN}- Norma{l}_{in- MGN}\right)\mid \end{array}} $$

In these two equations, *Score*_*dif*_ represent difference between the expression level of the IIMATs between the samples with MGN and the matched normal controls. *P*_*immune*_, *P*_*inflammation*_ and*P*_*MGN*_ represent the *P*-values of immune, inflammation and MGN-related genes, respectively, for each IIMAT derived from the above t-test between MGN and normal samples. Score_PCC_ represents the absolute distinction of the PCC score between the MGN patients and matched normal samples for each IIMAT. MGN_PCC_ and Normal_PCC_ refer to the PCC values of the immune-inflammation, immune-MGN and inflammation-MGN interaction pairs for the MGN and matched normal samples, respectively. Combined scores of each IMMAT was calculated using an equally weighted method based on above two integrated scores [20]. This multidimensional rank method could integrate two independent scores to a last ranking score. Higher ranking scores represent stronger dysregulated level of IIMATs between the MGN patients and matched normal samples. Randomly disturbing all sample labels 1000 times for the gene expression profile is used to generate permutation-based final ranking scores. Significant *P*-values (*P* < 0.05) are obtained by comparing the final ranking score with permutation-based final ranking score for each IIMAT. All above statistical analyses were performed using the R software (version 3.2.3; https://www.r-project.org/). The dysregulated IIMAT network was constructed based on these dysregulated IIMATs in MGN.

### Identification and classification power of core clusters from dysregulated IIMATs network for MGN

We used ClusterOne package in cytoscape with default parameters (http://apps.cytoscape.org/apps/ClusterONE) to identify core clusters from dysregulated IIMATs network. At last, we obtain four core clusters follow cluster scores from ClsuterOne. The gene expression data for each core cluster is used to classify MGN and control normal samples based on consensus clustering method [21]. Consensus clustering emerges as a promising solution to find cluster structures from data. As an efficient approach for consensus clustering, the K-means based method has garnered attention in the literature. We use ConsensusClusterPlus package in R (https://www.r-project.org/) to perform this process. The best category number is defined as the smallest increase in the area under the cumulative distribution function (CDF). Combining the classification result of the consensus clustering and the real category (disease and control) of the samples, we use chi-square test to discovery the association between the two classification methods. Finally, we use chi-square test to estimate if the core cluster could classify MGN and control normal samples (*P* < 0.05).

### Identification of drug repurposing candidates for MGN based on IIMATs

The gene-drug interaction data are download from DrugBank (https://www.drugbank.ca, Reversion 5.1.1, 2018-07-03) which is a public database including comprehensive molecular information about drugs, their mechanisms, their interactions and their targets [22]. Gene-drug interactions were got from DrugBank. Then a drug-related dysregulated IIMATs network is constructed and analyzed to identify drug repurposing candidates for MGN.

### Functional enrichment analysis for dysregulated IIMATs in MGN

Genes in dysregulated IIMATs are extracted for functional enrichment analysis to represent the functions. Online Enrichr tool is applied with default parameters to functional enrichment analysis [23]. Significant enriched GO terms (*P* < 0.01) and KEGG pathways (P < 0.01) are selected.

## Results

### The construction of global IIMATs network and topological analysis

**Fig. 1:**
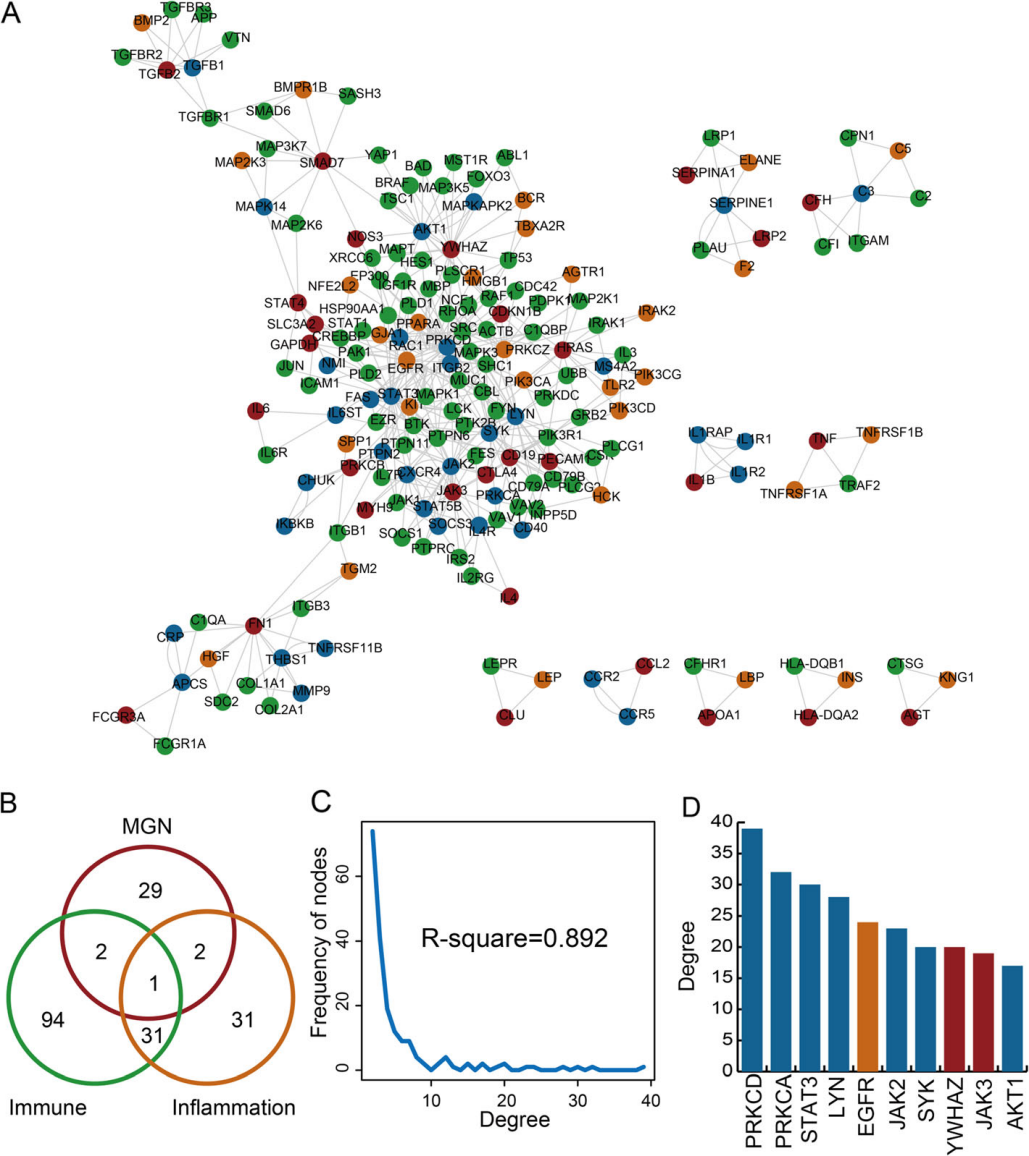
Construction and global characteristics of the IIMAT network for MGN. **a** A global IIMAT network for MGN. Red, green, yellow and blue represent MGN-, immune, inflammation and multifunction-related genes, respectively. **b** The venn diagram shows the common genes between three kinds of genes in IIMAT network. **c** Degree distribution of all nodes in the IIMAT network. **d** The degrees of nodes with the highest degrees in the IIMAT network

A global IIMATs network is constructed based on immune response, inflammation and MGN-related genes and protein and protein interactions (Fig. 1a). The whole IIMAT would be removed when the same gene play two different roles in a IIMAT. The global IIMATs network included 274 IIMATs, 190 nodes (94 immune, 31 inflammation, 29 MGN, 31 inflammation&immune, two MGN&immune, two MGN&inflammation and one MGN&inflammation&immune-related genes) and 478 edges (Fig. 1b). The global IIMATs network exists a scale-free distribution (R-square = 0.892) (Fig. 1c). The scale-free distribution is a specific topological feature of transcriptional regulatory network. Moreover, we also discovered some genes with high degree in global IIMATs network (Fig. 1d). The gene with highest degree is PRKCD (degree = 39). Gene PRKCD is only inflammation, immune and MGN-related gene in the IIMATs network. These high degree genes are almost immune or inflammation-related genes. It indicates that immune response and inflammation-related genes play essential roles in the whole network. These above comprehensive results indicated that global IIMATs network could be a useful background for studies of MGN.

### Some IIMATs were dysregulated in MGN patients

**Fig. 2:**
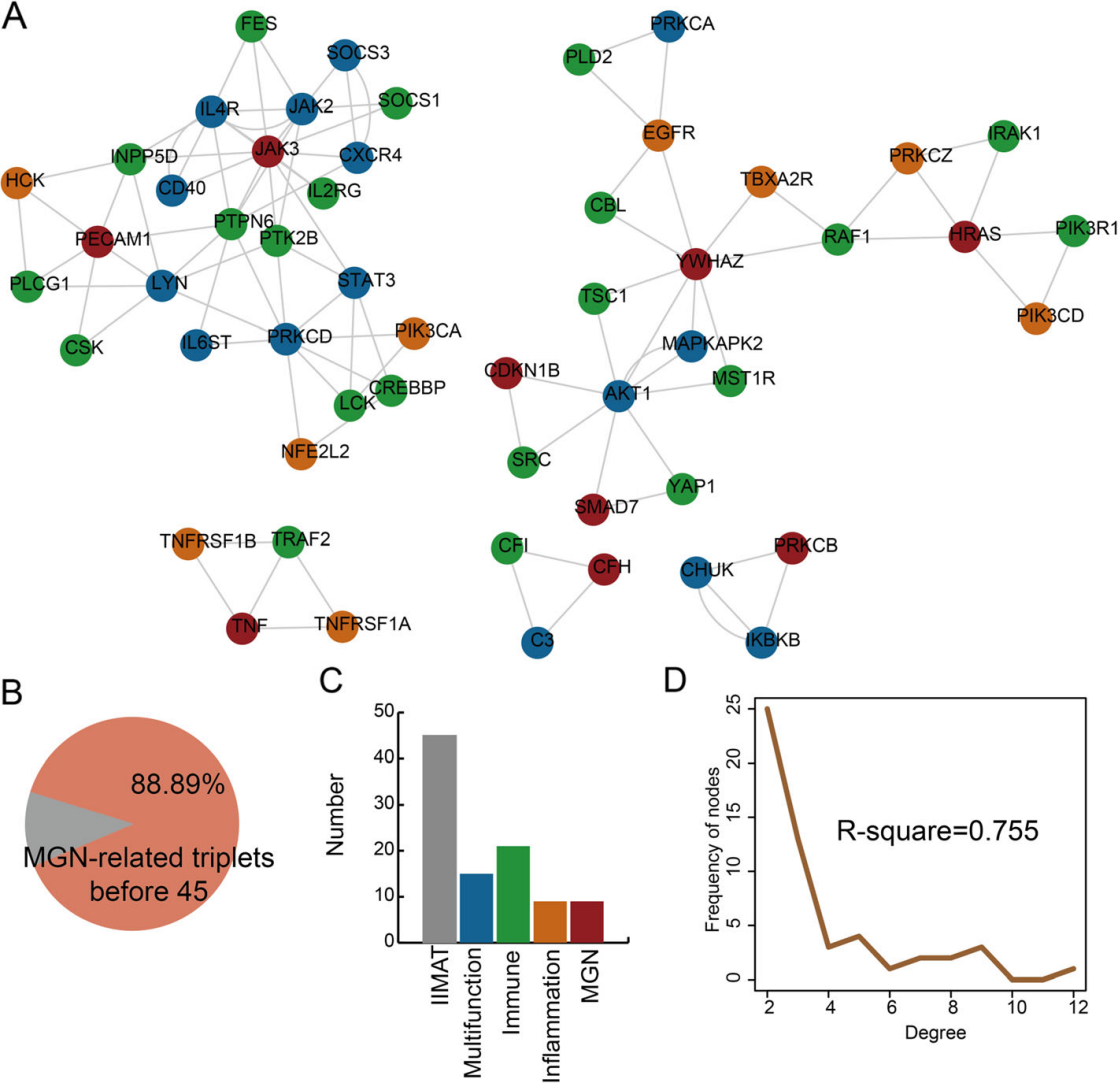
The significantly dysregulated IIMATs for MGN from global IIMAT network. **a** The significantly dysregulated IIMAT network for MGN. **b** Pie charts showing the percentages of significantly dysregulated IIMATs out of all IIMATs in the global IIMAT networks. **c** The numbers of IIMATs, multifunction-, immune-, inflammation- and MGN-related genes in the dysregulated IIMAT network. **d** Degree distribution of all nodes in the dysregulated IIMAT network for MGN

A comprehensive computational approach is developed to identify significantly dysregulated IIMATs in MGN patients. 45 dysregulated IIMATs are identified in MGN by above method. The detailed scores and *P*-values of permutation were listed in Additional file 3: Table S3. A dysregulated IIMATs network for MGN is constructed and the network contained 54 nodes and 98 edges (Fig. 2a). There were 88.89% significant IIMATs rank before 45 in all the dysregulated IIMATs (Fig. 2b). This result indicates that this computational approach accurately identified some dysregulated IIMATs for MGN. There are 45 IIMATs, 15 multifunctional, 21 immune-related, 9 inflammation-related and 9 MGN-related genes in this dysregulated network for MGN (Fig. 2c). Moreover, this network still show a scale-free degree distribution (Fig. 2d). These comprehensive results indicate that the significantly dysregulated IIMATs network showed closer network structure features and can be a functional network for MGN.

### Risk score profiles and multiple dysregulated patterns of IIMATs for MGN patients

**Fig. 3:**
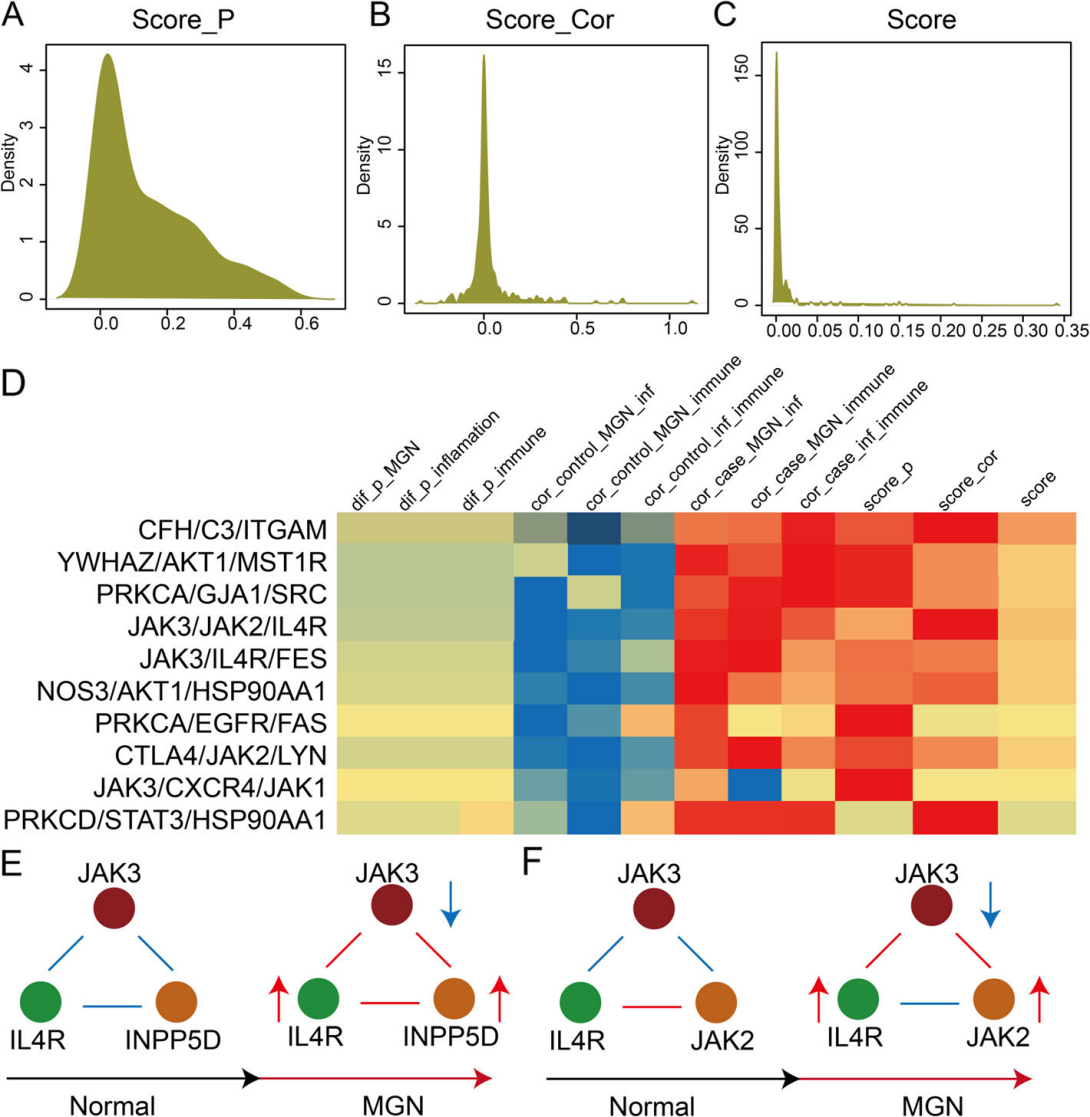
Construction of an activity score profile for IIMATs in the dysregulated IIMAT network of MGN. **a**-**c** The density distribution curves of the Scoredif, ScorePCC and integrated score. **d** The top ranked IIMATs with the highest scores in the dysregulated IIMAT network of MGN. The heat map shows the multiple scores of each dysregulated IIMAT in MGN. **e**-**f** The dysregulated patterns of two IIMATs in MGN. The red and blue lines represent positive and negative correlation between two kinds of genes. The red and blue arrows represent up- and down-regulated

We use risk score profiles to depict the compactness of three kinds of genes in each dysregulated IIMAT. The Score_dif_, Score_PCC_ and global score distributions are constructed and they all concentrated in a small scale (Fig. 3a, b, c). They also show similar unimodal distribution. The detail risk score profile of top ten dysregulated IIMATs for MGN are also shown (Fig. 3d). We found only individual or some risk scores change between normal samples and MGN patients in most IIMATs. For instance, the change level of Score_dif_ in IIMAT CFH/C3/ITGAM between normal sample and MGN patient is smaller than Score_PCC_. For the IIMAT JAK3/CXCR4/JAK1, only the Score_dif_ occur significant change in MGN patients. Thus we inferred that the patterns of these dysregulated IIMATs in MGN patients were complex and multiple. In dysregulated IIMAT JAK3/IL4R/INPP5D, the interactions between all genes are changed from negative correlation to positive correlation in MGN patients. MGN-related gene JAK is down-regulated and immune response- and inflammation-related genes IL4R and INPP5D are up-regulated in MGN patients (Fig. 3e). For another dysregulated IIMAT JAK3/IL4R/JAK2 (Fig. 3f), the interactions between JAK3 and IL4R, JAK3 and JAK2 are changed from negative correlation to positive correlation. The interaction between IL4R and JAK2 is changed from positive correlation to negative correlation. The genes in this IMMAT are all dysregulated. Collectively, the interactions between genes in dysregulated IMMATs are destroyed or rebuilt in MGN patients and indicate complex characteristics.

### MGN-related core IMMATs clusters could as specific biomarker to distinguish MGN and matched normal samples

**Fig. 4:**
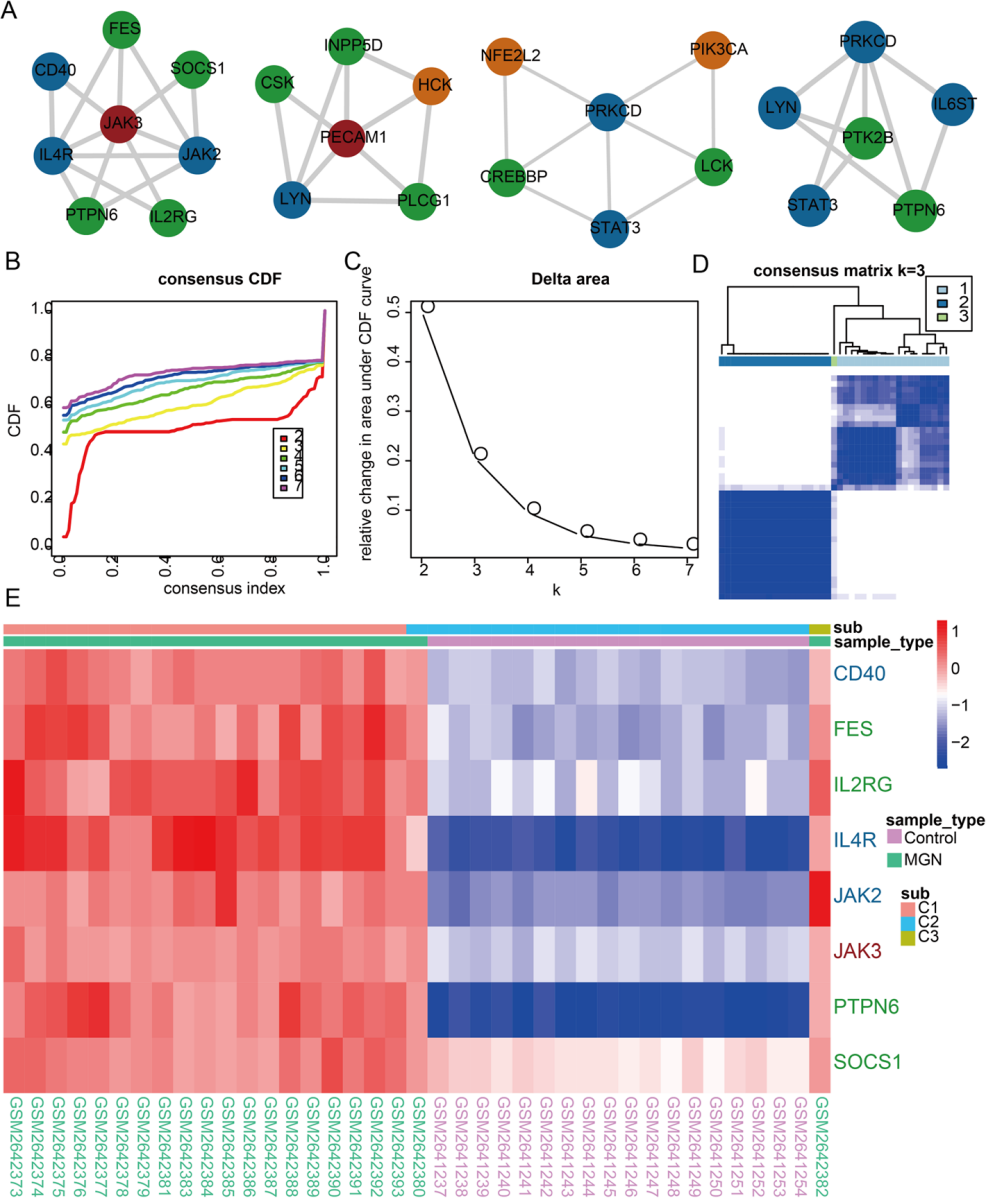
The core clusters could distinguish MGN patients and control normal samples. **a** The core clusters extracted form dysregulated IIMAT network of MGN. **b** Cumulative distribution function plot of the consensus index. **c** Relative change in area under CDF curve of different group number. **d** Consensus cluster heat map of all samples. **e** The gene expression heat map, sub label refers to the group type classified by consensus cluster method, and sample type refers to the disease status of the samples

We perform cluster analysis to explore the communication between MGN-, immune response- and inflammation-related genes in dysregulated IIMATs network for MGN. Four core clusters are extracted from dysregulated IIMATs network and each core cluster contained a certain number of MGN-, immune response- and inflammation-related genes (Fig. 4a). A consensus clustering approach is used to evaluate if core clusters could classify MGN patients and normal samples based on gene expression profile. Take the first core cluster for instance, the first core cluster could distinguish all samples into diverse groups follow consensus clustering method. We generally consider two factors including CDF and relative change in area under CDF curve plot to decide the final number of group is three (Fig. 4b and c). We discovered that each group has a consensus expression pattern and could distinguish clearly with other groups (Fig. 4d). Most MGN and normal samples could be distinguished accurately for these 3 groups (Chi-square test, *P* value = 2.286e-08) (Fig. 4e). We found only one MGN sample GSM2642380 has a wrong grouping and other MGN samples are all divided into subgroup one and three. All control samples are all divided into subgroup two. These comprehensive results showed that core clusters extracted from dysregulated IIMATs network could be served as specific biomarkers to discriminate MGN patients from normal samples.

### Identification of novel drug repurposing candidates for MGN based on IIMATs

**Fig. 5:**
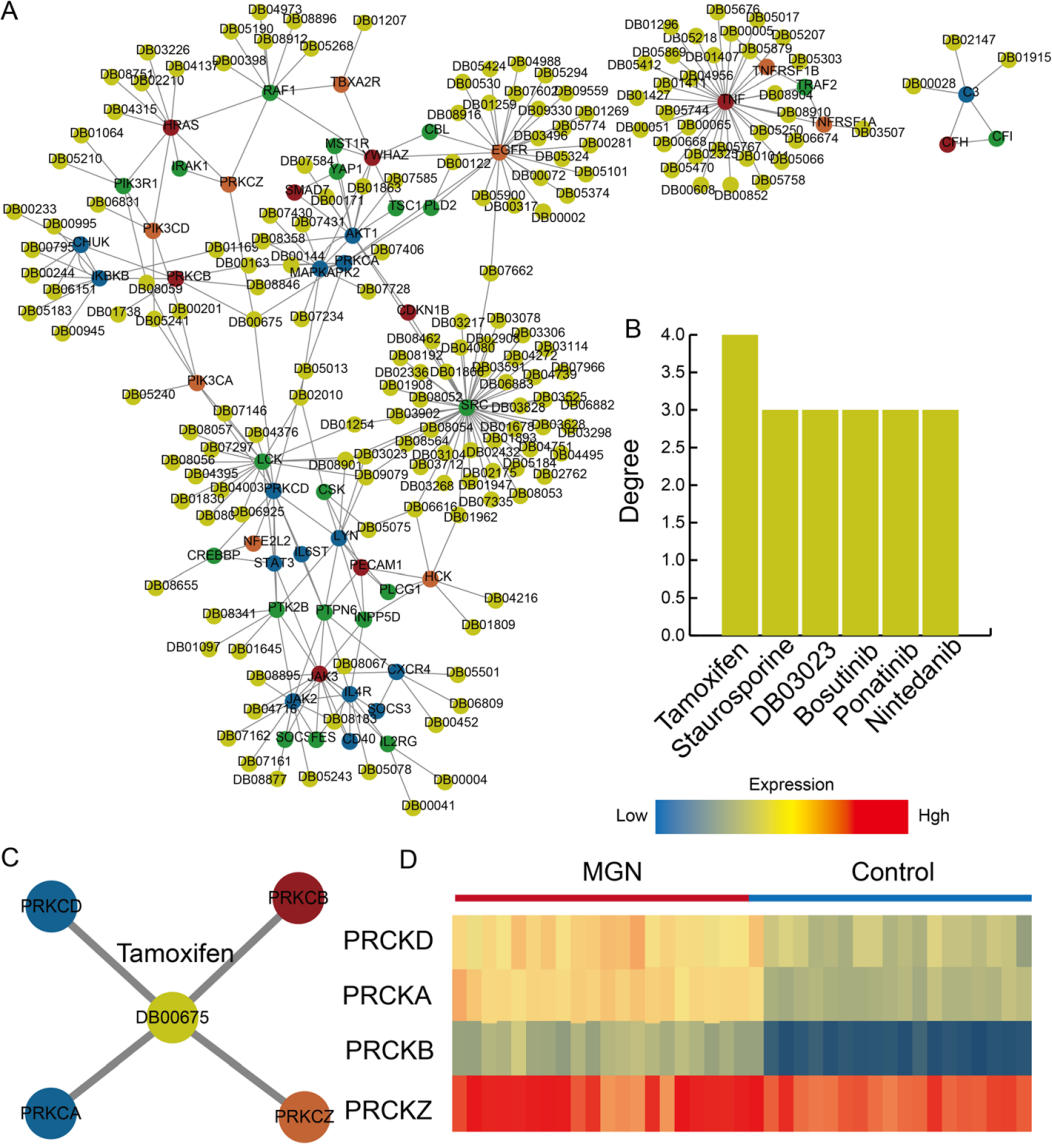
Drug repurposing candidates for MGN based on dysregulated IIMATs. **a** The drug-related dysregulated IIMAT network for MGN. **b** The drugs with high degree in the drug-related dysregulated IIMAT network. **c** The Tamoxifen-related sub-network extracted from drug-related dysregulated IIMAT network. **d** The heat map shows the expression of genes in Tamoxifen-related sub-network

In order to obtain novel drug repurposing candidates for MGN, we construct a drug-related IIMATs network based on drug-target genes and dysregulated IIMATs network. The drug-related IIMATs network contained 230 nodes (8 MGN-, 21 immune-, 9 inflammation-, 15 multifunction-related genes and 177 drugs) and 302 edges (Fig. 5a). The drugs with highest degree are Tamoxifen, Staurosporine, DB03023, Bosutinib, Ponatinib and Nintedanib (Fig. 5b). Most of these drugs are anti-cancer drugs. Some previous studies reveal that MGN may occur in association with drug use or systemic diseases such as infections and cancer [24]. Thus we inferred that some anti-cancer drugs maybe could serve as novel drug repurposing candidates for MGN. We further analyzed the drug Tamoxifen which is a drug shows the highest degree. Tamoxifen is a kind of anti-cancer drug which major treatment breast cancer and is also being studied for other types of cancer [25, 26]. In present drug-related IIMATs network, there are four genes including PRKCD, PRKCB, PRKCA and PRKCZ interact with tamoxifen and they are all differential expressed between MGN patients and normal samples (Fig. 5c and d). These four genes are all belong to protein kinase C (PKC) family which is a family of serine- and threonine-specific protein kinases that can be activated by calcium and the second messenger diacylglycerol. PKC family members phosphorylate a wide variety of protein targets and are known to be involved in diverse cellular signaling pathways. Some previous studies reported that the treatment effect of tamoxifen on MGN [27]. In addition, a previous study suggests that nintedanib may be a potential trigger for MGN [28]. Nintedanib is a multiple tyrosine kinase inhibitor that works on key angiogenesis pathways including platelet-derived growth factor, vascular endothelial growth factor, and basic fibroblast growth factor. Collectively, our results suggest that some anti-cancer drugs could be candidate drugs for MGN based on the computational drug repurposing method.

### Dysregulated IIMATs for MGN are associated with critical biological functions and the chemokine signalling pathway

**Fig. 6:**
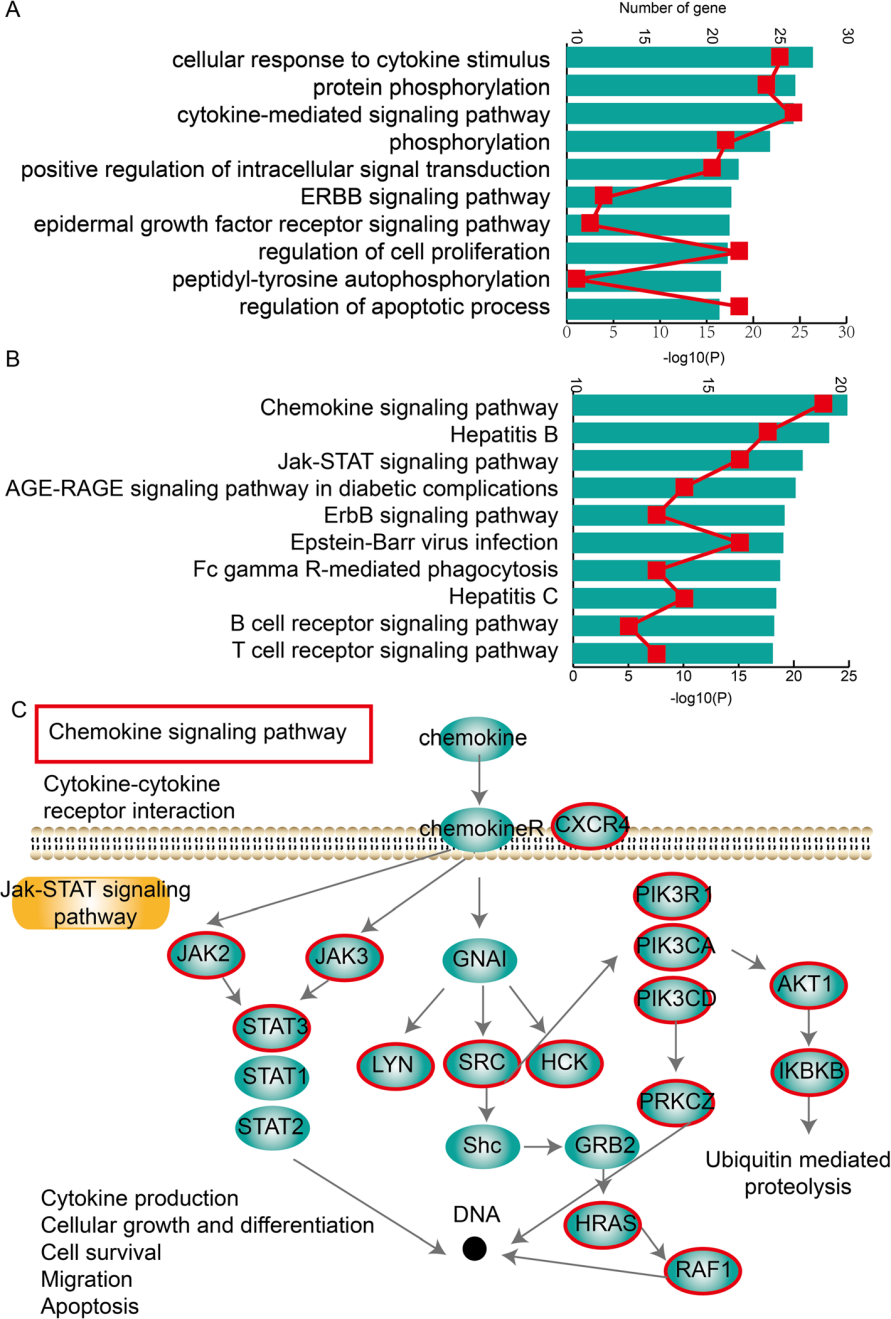
Functional analysis of genes in dysregulated IIMATs for MGN. **a** GO terms enriched for genes in dysregulated IIMATs for MGN, ranked by −log10(P) are presented as bar plots. The red lines represent the number of enriched genes. **b** KEGG pathway enriched for genes in dysregulated IIMATs for MGN, ranked by −log10(P) are presented as bar plots. **c** The part of Chemokine signaling pathway was shown and participant genes in dysregulated IIMATs are also shown

The GO terms analysis is performed based on the genes in dysregulated IIMATs network. We find these genes are enrichment in GO terms such as cellular response to cytokine stimulus, protein phosphorylation, cytokine-mediated signaling pathway and so on (Fig. 6a). The KEGG pathway analysis is also performed and some key pathways such as chemokine signaling pathway, JAK-STAT signaling pathway, B/T cell receptor signaling pathway and so on (Fig. 6b). Specially, we focus on a key pathway called chemokine signaling pathway (Fig. 6c). Chemokines are a family of small cytokines, or signaling proteins secreted by cells and some of them are considered pro-inflammatory and can be induced during an immune response to recruit cells of the immune system to a site of infection. The cytokine-cytokine receptor interaction and JAK-STAT pathway are essential parts of chemokine signaling pathway. They are all essential for immune and inflammatory responses [29]. In our analysis, we discover that several genes in dysregulated IIMATs network for MGN play essential roles in this pathway, which indicated that these key genes identified by us were highly associated with the MGN, immune response and inflammation.

## Discussion

The present study focuses on the interactions among MGN-, immune- and inflammation-related genes based on an integrated computational approach using PPI network and gene expression profile. The essential roles of immune- and inflammation-related genes in the occurrence, development and treatment of MGN have been reported in large number of studies [30, 31]. However, there is not global and system method for identification of biomarkers and drug repurposing candidates of MGN based on IIMATs. Thus we develop a computational and comprehensive method to identify MGN-related IIMATs and perform drug repurposing for MGN.

The immune- and inflammation-related genes play essential roles in development and treatment of MGN [32, 33]. In our study, we explore the relations among immune-, inflammation- and MGN-related genes and these three kinds of genes could form an IIMAT to contribute to the development and treatment of MGN. We find these three kinds of genes show close associations on network structure and expression level. These dysregulated IIMATs also could serve as the biomarker to distinguish MGN patients and normal samples.

More and more novel targeted therapies are being developed because of increasing knowledge of the molecular mechanisms underlying many kinds of diseases. It’s unclear that the rarer side effects from these relatively newer agents and few dedicated studies are available. Thus drug repurposing is an effective method to rediscover a new indication for an existing drug. The advantages of drug repurposing are less time-consuming and expensive and reduced risk of unexpected side effects. In our analysis, we discover some anti-cancer drugs such as tamoxifen and nintedanib maybe influence MGN based on our computational approach. Previous studies provide epidemiologic evidence of an excess of cancer risk in patients with MGN [34–36]. However, the relationships of mechanism between MGN and cancer remain unclear. Follow the result of drug repurpsoing, we infer that MGN and cancer construct relations based on immune and inflammation response. This study provided candidate biomarkers and drugs for MGN, validation of the candidate genes and drugs by biological experiments warrants detailed studies in the future. As more large-scale molecular profiles and clinical data of MGN become available, it could improve robustness and predictive capacities of our approach.

## Conclusions

Overall, we construct an IIMATs network for MGN and identify some dysregulated IIMATs. The dysregulated pattern of these dysregulated IIMATs are complex and various. Then we identify some core clusters from dysregulated IIMATs network in MGN and the core cluster could distinguish MGN patients and normal samples. Specially, we perform a computational drug repurposing approach to identify novel drug candidates for MGN. Functional analysis shows that dysregulated IIMATs are associated with some key functions and chemokine signaling pathway. Collectively, our results contributed to better understanding of the relationships among immune-, inflammation- and MGN-related genes and identify promising drug repurposing candidates for MGN.

## Supplementary information

**Additional file 1: Table S1.** Immune, inflammation and MGN-associated genes

**Additional file 2: Table S2.** Baseline characteristics for MGN

**Additional file 3: Table S3.** Scores of dysregulated IMMATs for MGN

## Data Availability

All data generated or analysed during this study are included in this published article Additional file 1: Table S1, Additional file 2: Table S2 and Additional file 3: Table S3. The Gene Expression Omnibus (https://www.ncbi.nlm.nih.gov/geo/) datasets analyzed during this study are under the following accession GSE99340 [19].

## Abbreviations

CDF: Cumulative distribution function
HPRD: Human Protein Reference Database
IIMATs: Immune-, inflammation- and MGN-associated triplets
MGN: Membranous glomerulonephritis
NF-KB: Nuclear factor-kappa B
PCC: Pearson’s correlation coefficients
PPI: Protein-protein interaction
